# Comparison of dynamic changes in stressed intravascular volume, mean systemic filling pressure and cardiovascular compliance: Pilot investigation and study protocol

**DOI:** 10.1371/journal.pone.0238045

**Published:** 2020-08-28

**Authors:** Konstantin Yastrebov, Laurencie Brunel, Zoe A. Williams, Hugh S. Paterson, Mariko Yata, Christopher S. Burrows, Innes K. Wise, Benjamin M. Robinson, Paul G. Bannon

**Affiliations:** 1 Department of Intensive Care, Prince of Wales Hospital, Sydney, Australia; 2 The University of New South Wales, Sydney, Australia; 3 DVC Research, University of Sydney, Sydney, Australia; 4 Royal Prince Alfred Hospital, Sydney, Australia; Azienda Ospedaliero Universitaria Careggi, ITALY

## Abstract

The mean systemic filling pressure (MSFP) represents an interaction between intravascular volume and global cardiovascular compliance (GCC). Intravascular volume expansion using fluid resuscitation is the most frequent intervention in intensive care and emergency medicine for patients in shock and with haemodynamic compromise. The relationship between dynamic changes in MSFP, GCC and left ventricular compliance is unknown. We conducted prospective interventional pilot study following euthanasia in post cardiotomy adult sheep, investigating the relationships between changes in MSFP induced by rapid intravascular filling with fluids, global cardiovascular compliance and left ventricular compliance. This pilot investigation suggested a robust correlation between a gradual increase in the intravascular stressed volume from 0 to 40 ml/kg and the MSFP r = 0.708 95% CI 0.435 to 0.862, making feasible future prospective interventional studies. Based on the statistical modelling from the pilot results, we expect to identify a strong correlation of 0.71 ± 0.1 (95% CI) between the MSFP and the stressed intravascular volume in a future study.

## Introduction

Intravascular volume expansion using fluid resuscitation is the most frequent intervention in intensive care and emergency medicine for patients in shock and with haemodynamic compromise. The estimation of the fluid status in clinical practice remains imprecise and lacks a gold standard [1]. There is major variability in recommendations regarding the amount of intravenous fluid to be administered in different clinical scenarios and patient groups. The World Health Organization estimates that severe sepsis and septic shock is responsible for over 6 million deaths worldwide each year. Recommendations for fluid resuscitation in septic shock suggest rapid intravenous administration of crystalloid boluses up to 30 ml/kg in adults [2] and up to 40–60 mL/kg in children (10–20 mL/kg per bolus) [3]. However, mounting evidence also indicates that injudicious administration of intravenous fluids increases mortality and morbidity [4,5].

The Mean systemic filling pressure (MSFP) represents an interaction between the intravascular volume and the global cardiovascular compliance (GCC) [6, 7]. Its major physiological purpose appeared to be the provision of a driving pressure for venous return being the difference between MSFP and right atrial pressure in functioning heart. MSFP is generated by the elastic forces of global cardiovascular system exerted against the stressed volume of blood. The unstressed volume of blood is a proportion of the total blood volume which is required to fill the cardiovascular system to zero atmospheric pressure. The stressed volume of blood is the total blood volume minus the unstressed volume. The stressed volume of blood exceeds the unstretched capacity of the cardiovascular system and depending on the intrinsic compliance of the cardiovascular system, is ultimately responsible for the generated pressure. When the circulation stops, there is equalization of pressures within multiple cardiovascular compartments but with differences in compliance. This residual static pressure is the MSFP. In live animals cardiovascular compliance is highly dynamic between the compartments due to the active contractile properties of the smooth muscles within vascular walls and involuntary striated muscles of the heart. There is an additional component of external forces applied to the cardiovascular system, which is generated by the functioning organs and tissues. The combination of these active forces disappears after death, prior to the onset of rigor mortis and shifts of fluid from the intravascular compartment, thus presenting an ideal short period in time to measure MSFP. MSFP differs between different species of animals and humans. To our knowledge physiological levels of MSFP in sheep have not been investigated.

There is currently no gold standard for the routine measurement of the mean systemic filling pressure in clinical practice [8–10]. Equilibration of pressure within cardiovascular system following cardiac arrest represents the most accurate measurement of MSFP but it is not applicable to patient management. Cardiovascular compliance is highly dynamic in live patients, being largely affected by active vascular and cardiac contractions which further complicate estimations of MSFP. Recent human research suggested an analogue for mean systemic filling pressure P_msa_ as a clinically acceptable surrogate for MSFP [11]. Establishing the physiological level of MSFP in different animal species and changes in the MSFP following variable volumes of fluid therapy may improve targets for fluid resuscitation in veterinary medicine and serve as a translation research guide in future human investigations.

We hypothesised that there is a definable non-linear relationship between changes in cardiovascular compliance and the mean systemic filling pressure, induced by alterations in the stressed intravascular volume in sheep.

## Materials and methods

We are planning an interventional study in adult merino sheep used for research in the veterinary hybrid operating theatre at the University of Sydney, Australia. The study will be an extension of projects previously approved by the University of Sydney (Australia) Animal Research Ethics Committee (2019/1650 amendment) and will be conducted at the Charles Perkins Centre for Research, The University of Sydney (Sydney, Australia). The investigation will be performed in accordance with the Helsinki Convention guidelines for humane care of animals. After consultation with the Ethics Committee, it was further determined that the pilot and the future MSFP studies do not require specific approval because they are performed on deceased animals. The study protocol and statistical analysis plan were modelled based on the completed pilot investigation and were finalised before data collection was initiated.

### Pilot investigation

#### Subjects

Seven female adult sheep (2 years old, mean weight 49.4 ± 3.1 kg) were euthanized under general anaesthesia by exsanguination into the cardiopulmonary bypass circuit following open chest mitral valve surgery. Exsanguination resulted in 0 mmHg arterial and venous pressures and cardiac arrest. All sheep initially received premedication with methadone and midazolam, followed by general anesthesia induced with propofol and maintained with isoflurane. They required intraoperative invasive monitoring of arterial pressure, central venous pressure and cardiac output as part of the routine protocol for the premortem study and received fluid resuscitation during the intraoperative period. The heart was exposed via a left thoracotomy. Cardiopulmonary bypass was established with right atrial and aortic cannulation. Mitral interventions were performed on a normothermic beating heart via a left atriotomy. Sheep were then euthanised in accordance with the protocol by rapid exsanguination into the cardiopulmonary bypass circuit until the arterial and central venous pressures reached 0 mm Hg. Cardiac arrest was confirmed visually, by the complete loss of electrocardiographic activity and by the loss of the arterial pulsatile pressure waveform.

### Post-mortem intervention

The blood removed during exsanguination was returned from the cardiopulmonary bypass circuit via aortic cannula into the circulation. Four rapid boluses 10 ml/kg of crystalloid solution were then sequentially administered via the aortic cannula to further increase the intravascular volume. Pressures in the arterial and central venous access points were allowed to equalize after each intravascular volume manipulation.

### Measurements

All sheep had an arterial pressure catheter placed in the aortic arch (Arrow, CS-15703-E, 7Fr 20 cm triple lumen central line (Teleflex 6Fr sheath introducer), a central venous catheter placed in the left internal jugular vein (Arrow, CS-15703-E, 7 Fr 20 cm triple lumen central line. Reading, Pennsylvania USA) and a Transonic Two Channel Perivascular Flowmeter (Model T402) with a ‘COnfidence Flowprobe’ (Ithaca, NY 14850 USA) placed on the main pulmonary artery trunk, as determined by the protocol. Arterial pressure, central venous pressure and cardiac output were continuously recorded up until the cardiac arrest as per the original research protocol (Philips Patient Monitor, IntelliVue MX800 (Philips Medizin Systeme Boeblingen GmbH, Hewlett-Packard-Str.2 71034 Boeblingen, Germany).

The exsanguinated volume was recorded when arterial and central venous pressures reached 0 mmHg and was deemed to represent the “stressed” blood volume at the time of death.

Rapid reinfusion of the stressed volume from cardiopulmonary bypass circuit back into circulation and the bolus infusions of the crystalloid solution were administered via the aortic perfusion cannula and each was followed by a period of several minutes for equalization of pressures in arterial and central venous points of measurements at which stage the pressure was recorded as a “mean systemic filling pressure”. The equalization pressure recorded after reinfusion of the stressed blood volume was deemed to represent the mean systemic filling pressure at the time of death.

It was previously suggested that the analogue mean systemic filling pressure (P_msa_) can be calculated according to the Eq 1 [12]:

$${\text{P}}_{\text{msa}}=(0.96\times \text{CVP})+(0.04\times \text{MAP})+(\text{c}\times \text{CO})$$ (1)

where CVP is central venous pressure, MAP is mean arterial pressure, CO is cardiac output and c is a factor to adjust the influence of resistance to venous return according to the patient’s age, height and weight [9]. Factor c in humans was defined as (Eq 2):

$$\text{c}=0.038\times (94.17+0.193\times \text{age})/(4.5\times [0.9^{\left(\text{age}-15\right)}]\times 0.007184\times [{\text{height}}^{0.725}]\times [{\text{weight}}^{0.425}])$$ (2)

The formula for calculation of factor c in an ovine model is not known. We calculated factor c by rearranging the Eq 1 and populating it with known values at the time of death. As factor c is a constant individual anthropometric value, it was then used in Eq 1 to calculate P_msa_ using recorded invasive haemodynamic data prior to the cardiopulmonary bypass.

The pressure gradient for venous return was calculated by the difference between mean systemic filling pressure and central venous pressure.

A qualified echocardiographer (Advanced Transthoracic Echocardiography training, Level 3 [13] or a certified veterinary echocardiographer) performed the echocardiographic measurements (SC2000, Siemens Healthcare GmbH, Erlangen, Germany echocardiography scanner with 10V4 MHz cardiac probe). Epicardial apical four and two chamber views were obtained in all cases using unfocused beam with 8.5 MHz scanning frequencies. Automatic left ventricular endocardial border tracing was used with manual adjustment as required. Left ventricular volumes were estimated at each volume state using Simpson’s technique and averaged between four and two-chamber views.

Global cardiovascular compliance was calculated according to the Eq 3:

GCVC=ΔVs/ΔMSFP (3)

where GCVC is global cardiovascular compliance, Δ Vs is induced change of stressed intravascular volume and Δ MSFP is a change of mean systemic filling pressure measured before and after the change in stressed volume.

Left ventricular compliance was calculated according to the Eq 4:

$$\text{LVC}=\Delta {\text{V}}_{\text{LV}}/\Delta \text{MSFP}$$ (4)

where LVC is left ventricular compliance, Δ V_LV_ is the change of the left ventricular volume induced by the alteration of stressed intravascular volume and Δ MSFP is a change of mean systemic filling pressure measured before and after the change in stressed volume.

### Statistical analysis

Normality of dependent variables was tested using the D’Agostino K-squared test. Continuous variables are expressed as the mean ± standard deviation (SD) or the median and interquartile range (IQR) as appropriate. Correlations were assessed by Pearson’s (r) and Spearman (rho) coefficients for normally and non-normally distributed variables respectively. Correlations were pre-defined as “moderate” or “weak” with correlation coefficients of less than 0.6 and 0.3 respectively. No corrections were made for multiple comparisons.

A ‘robust’ relationships between variables was pre-defined as those with statistically significant low levels of bias and imprecision and correlation coefficients > 0.7 where applicable.

Regression was used to derive equations and to determine the quadratic line of best fit for the global cardiovascular compliance at different fluid resuscitation volumes and for the left ventricular compliance at different levels of the mean systemic filling pressure. The analysis was performed using the data derived from all sheep experiments and separately with the sheep 3 and 4 excluded due to the violation of protocol in these two animals (inadvertent admix of air to the returned stressed volume).

Statistical analyses were performed using Stata 13 (College Station, Texas, USA).

## Results

A total of 7 sheep were investigated between October 2018 and August 2019. The animal characteristics are presented in Table 1. Haemodynamic variables are presented in Table 2.

**Table 1:** Sheep characteristics.

Sheep number	Weight (kg)	Age (years)	Sex
**1**	51	2	Female
**2**	54	2	Female
**3**	51	2	Female
**4**	50	2	Female
**5**	46	2	Female
**6**	45	2	Female
**7**	49	2	Female

**Table 2:** Haemodynamic characteristics of all animals.

Sheep number	SBP before CPB (mmHg)	DBP before CPB (mmHg)	MAP before CPB (mmHg)	CVP before CPB (mmHg)	HR before CPB (beats/min)	CO before CPB (l/min)	SBP immediately before the arrest (mmHg)	DBP immediately before the arrest (mmHg)	MAP immediately before arrest (mmHg)	CVP immediately before arrest (mmHg)	HR immediately before the arrest (beats/min)	CO immediately before the arrest (l/min)
**1**	102	87	94	15	105	3.829	69	48	54	16	124	2.521
**2**	65	54	59	7	101	3.784	67	52	59	8	96	3.009
**3**	69	49	54	14	107	2.919	79	55	62	12	94	2.901
**4**	69	51	58	16	78	3.773	75	41	54	17	91	2.162
**5**	68	55	59	10	128	2.998	59	31	42	13	142	1.675
**6**	67	48	54	18	131	2.854	62	33	42	21	104	1.720
**7**	68	51	56	16	94	3.666	53	29	35	11	116	2.339

Definition of abbreviations: CPB = cardiopulmonary bypass; SBP = systolic blood pressure; DBP = diastolic blood pressure; MAP = mean arterial pressure; CVP = central venous pressure; HR = heart rate; CO = cardiac output

### Intravascular volume

The mean drained “stressed” volume of blood was 2050 ± 210 ml, or 41.6 ± 4.2 ml/kg of the body weight. Individual results are presented in Table 3. Normal saline administered as four sequential 500ml boluses independently of individual weight produced a near-linear increase in MSFP in five sheep and resulted in inconsistent response in two sheep (number 3 and 4). Retrospective review of these two sheep indicated inadvertent admix of air to the returned stressed volume from cardiopulmonary bypass circuit.

**Table 3:** Stressed volume and mean systemic filling pressure measurements.

Sheep number	Blood volume drained (ml)	Stressed volume ml/kg	MSFP blood returned (mmHg)	MSFP 500 ml bolus (mmHg)	MSFP 1000 ml bolus (mmHg)	MSFP 1500 ml bolus (mmHg)	MSFP 2000 ml bolus (mmHg)
**1**	2000	39.2	23	26	29	31	33
**2**	2400	44.4	24	25	28	29	35
**3**	2100	41.2	17	20	16	16	24
**4**	1800	36	23	21	21	27	31
**5**	2100	45.6	19	20	22	26	29
**6**	2150	47.8	28	32	37	39	39
**7**	1800	36.7	17	21	24	27	28

Definition of abbreviations: MSFP = mean systemic filling pressure

### Pressure

The mean systemic filling pressure was 21.6 ± 4 mmHg following the return of the drained stressed volume from the cardiopulmonary bypass circuit back into the circulation. Individual measurements of the baseline MSFP and the MSFP measurements following administration of fluid boluses are presented in Table 3.

The correlation between MSFP and the stressed volume was r = 0.59, 95%CI 0.32–0.77. When sheep 3 and 4 were removed from the analysis due to attrition, the correlation between MSFP and the stressed volume was r = 0.71, 95%CI 0.44–0.86.

### Left ventricular volumes

The mean left ventricular volume was 14 ± 5 ml with circulation filled only by “non-stressed” volume of blood. The mean left ventricular volume was 43 ± 21 ml at the baseline filling with stressed volume. Individual measurements of left ventricular volume following administration of fluid boluses are presented in Table 4.

**Table 4:** Left ventricular volume measured with echocardiography.

Sheep number	LVEDV Echo before the arrest (ml)	LVESV Echo before the arrest (ml)	LV Volume after stressed volume drainage (ml)	LV Volume blood returned (ml)	LV Volume 500 ml bolus	LV Volume 1000 ml bolus	LV Volume 1500 ml bolus	LV Volume 2000 ml bolus
**1**	40	12	7	24	37	50	53	61
**2**	38	13	21	74	81	80	93	95
**3**	45	9	9	50	54	54	69	75
**4**	36	13	15	42	55	63	57	68
**5**	Missing data	Missing data	18	25	20	30	25	37
**6**	46	21	14	63	65	79	73	82
**7**	29	13	12	22	34	38	34	43

Definition of abbreviations: LVEDV = left ventricular end-diastolic volume; LVESV = left ventricular end-systolic volume; LV—left ventricular

The correlation between the left ventricular volume and the baseline cardiovascular stressed volume was r = 0.51, 95%CI 0.21–0.72. Excluding sheep 3 and 4, the correlation between the left ventricular volume and the baseline cardiovascular stressed volume was r = 0.49, 95%CI 0.12–0.74.

### Global cardiovascular compliance

**Fig 1:**
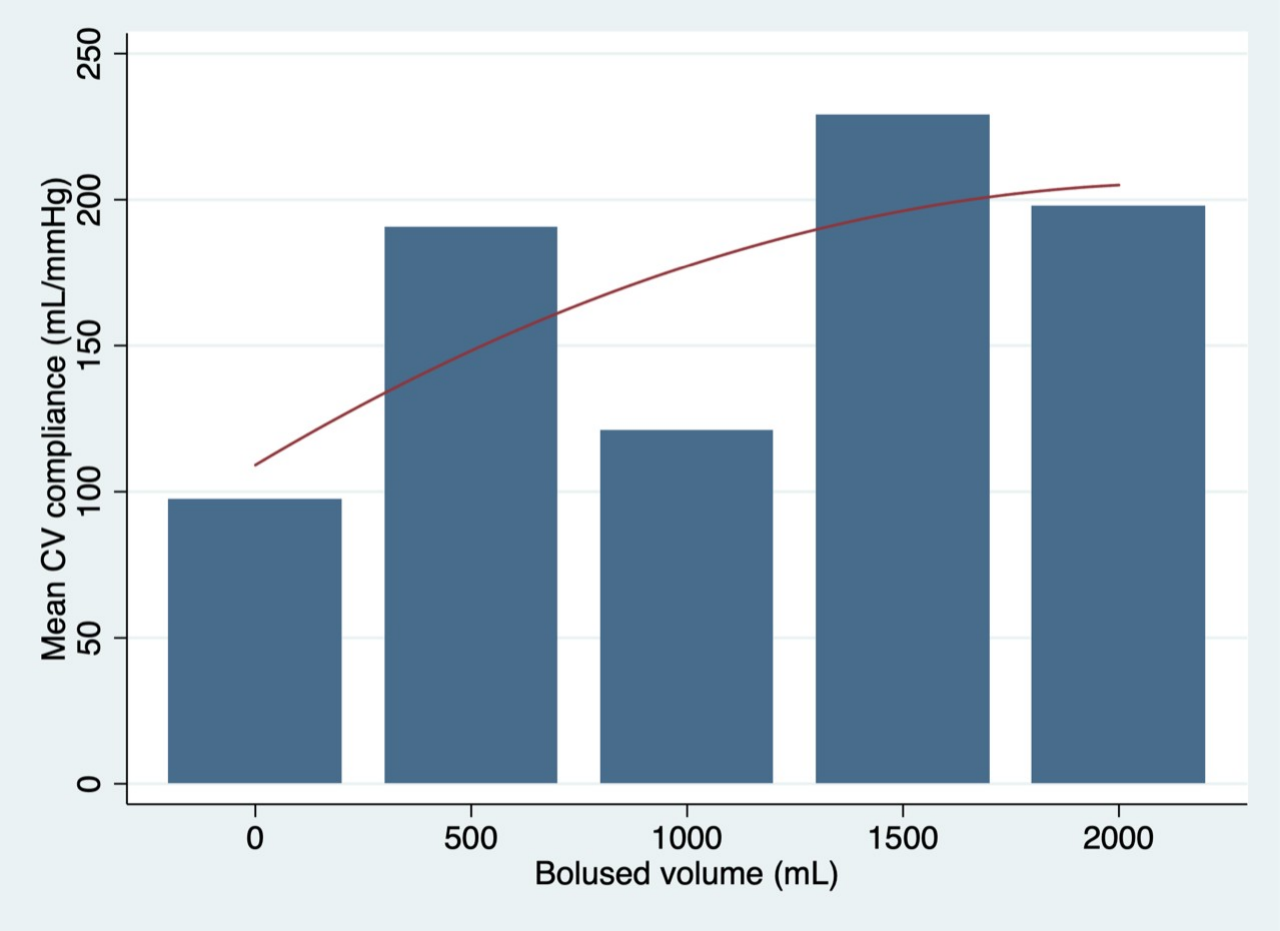
Graphic presentation of the mean global cardiovascular (CV) compliance plotted against bolus volume with a quadratic line of best fit with all sheep analysed.

The relationship between global cardiovascular compliance and bolused intravascular fluid volume with all sheep analysed is presented in Fig 1. The best equation to describe the trend of global cardiovascular compliance with all sheep included in the analysis was:

$$\text{Mean Global CV Compliance}=0.88\times \text{BV}-0.0001\times {\text{BV}}^{2}+109.$$

**Fig 2:**
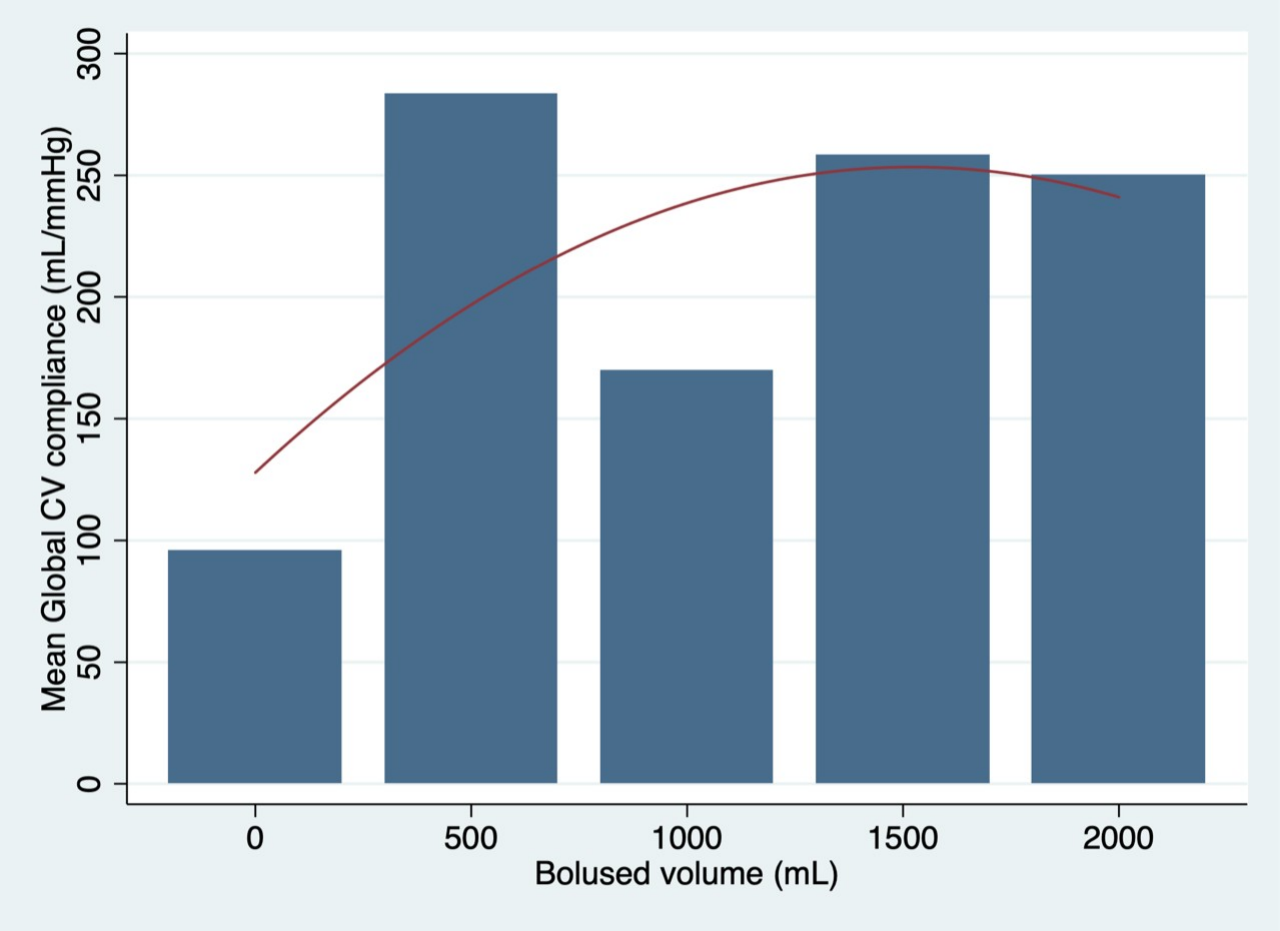
Graphic presentation of the mean global cardiovascular compliance plotted against bolus volume with a quadratic line of best fit with sheep 3 and 4 excluded.

The relationship between global cardiovascular compliance and bolused intravascular fluid volume with sheep 3 and 4 excluded from the analysis is presented in Fig 2. The best equation to describe the trend in global cardiovascular compliance with sheep 3 and 4 excluded was:

$$\text{Mean Global CV Compliance}=0.16\times \text{BV}-0.00005\times {\text{BV}}^{2}+128$$

### Left ventricular compliance

**Fig 3:**
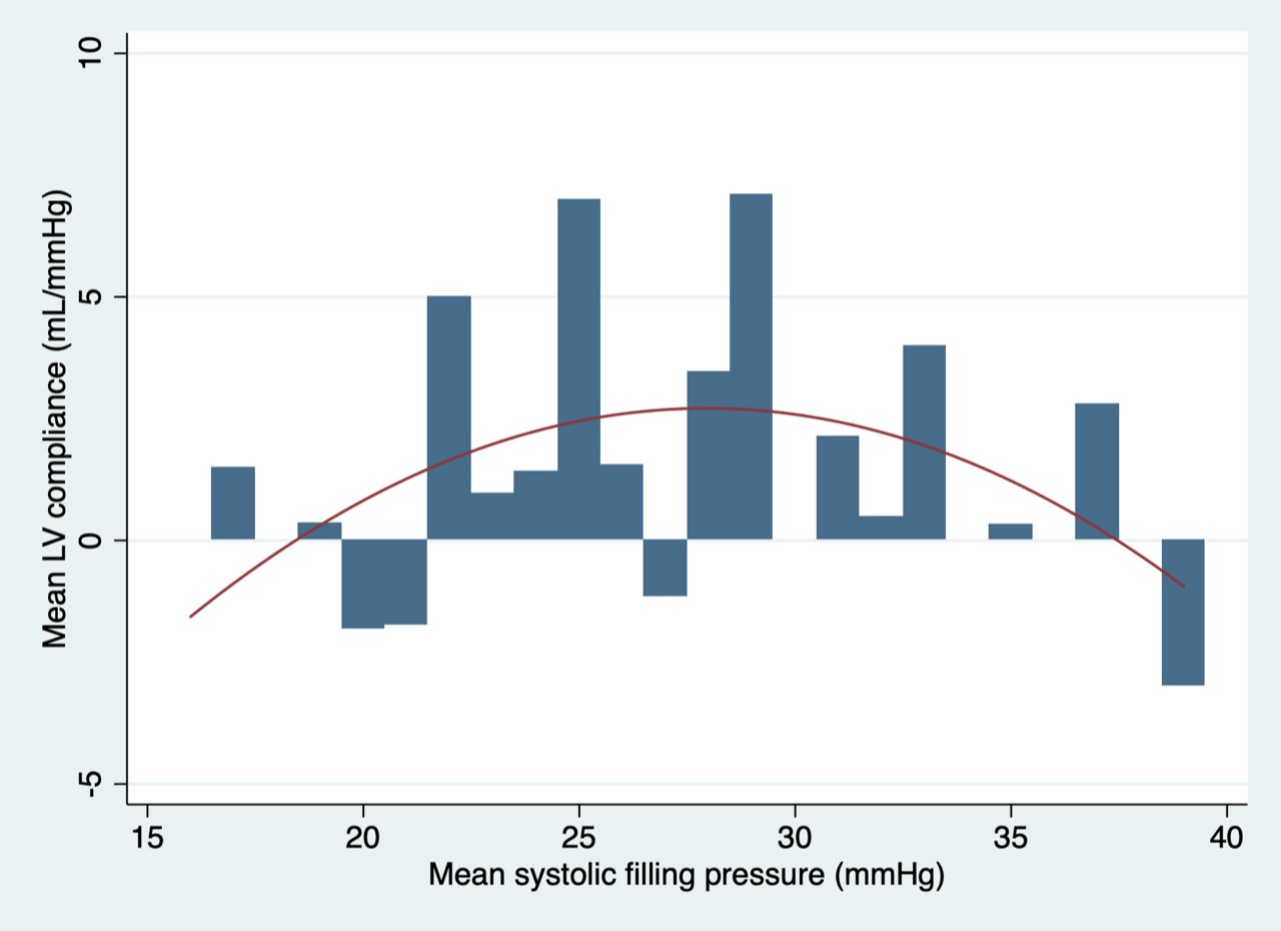
Graphic presentation of the mean LV compliance plotted against MSFP with a quadratic line of best fit with all sheep analysed.

The relationship between left ventricular compliance and the mean systemic filling pressure with all sheep analysed is presented in Fig 3. The best equation to describe the trend of left ventricular compliance with all sheep included in the analysis was:

$$\text{Mean LV Compliance}=1.68\times \text{MSFP}-0.3\times {\text{MSFP}}^{2}-20.7.$$

**Fig 4:**
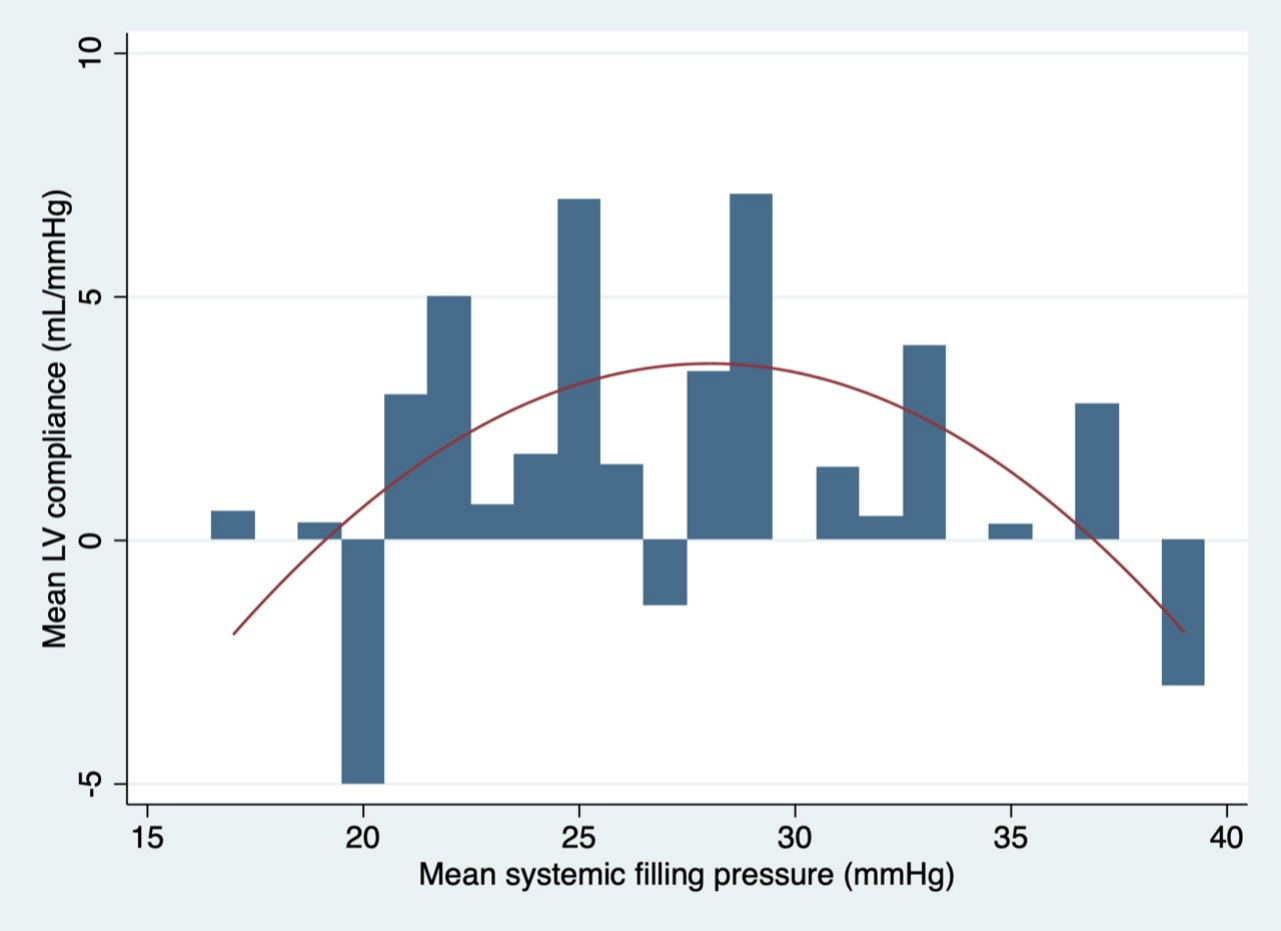
Graphic presentation of the mean LV compliance plotted against MSFP with a quadratic line of best fit with sheep 3 and 4 excluded from the analysis.

The relationship between left ventricular compliance and mean systemic filling pressure with sheep 3 and 4 excluded from the analysis is presented in Fig 4. The best equation to describe the trend of left ventricular compliance with sheep 3 and 4 excluded was:

$$\text{Mean LV Compliance}=2.65\times \text{MSFP}-0.46\times {\text{MSFP}}^{2}-32.25.$$

### Factor “c” and analogue mean systemic filling pressure (P_msa_)

The mean calculated factor “c” in this model was 2.65 ± 1.08. The mean analogue mean systolic filling pressure (P_msa_) calculated prior to the initiation of cardiopulmonary bypass was 24.7 ± 3.6 mmHg. Individual calculations of factor “c” and P_msa_ are presented in Table 5.

**Table 5:** Calculated factor “c” and P_msa_ prior to the cardiopulmonary bypass.

Sheep number	Factor “c”	Calculated P_msa_ prior to bypass
**1**	2.173741	28.30685
**2**	4.639415	19.1076
**3**	1.034126	23.33535
**4**	2.090657	27.67845
**5**	2.889552	19.9047
**6**	3.581395	27.0031
**7**	2.154767	27.3149

Definition of abbreviations: P_msa_ = analogue mean systemic filling pressure; factor “c” is a factor to adjust the influence of resistance to venous return according to the patient’s age, height and weight. The relationship between P_msa_ and factor “c” is described by the equation:

${\text{P}}_{\text{msa}}=(0.96\times \text{CVP})+(0.04\times \text{MAP})+(\text{c}\times \text{CO})$

## Discussion

Mean systemic filling pressures have been previously measured in intact rats [14], dogs [15] and pigs [16], producing values between 7 and 13 mmHg. MSFP has been measured in humans following cardiac arrest in intensive care [17] and estimated in various cohorts of patients in clinical settings [18–20]. In human patients the values varied between 14 and 24 mmHg depending on pre-existing fluid and vasopressor resuscitation. The relatively high values of MSFP and P_msa_ in our study are likely to reflect intensive volume resuscitation and high dose vasopressor infusions administered during the premortem study including immediately prior to the induced cardiac arrest. It is likely that the blood returned from CPB contained significant amounts of adrenaline, noradrenaline and vasopressin.

The inadvertent intra-aortic insufflation of air likely attributed to the lower correlation found between the MSFP and the stressed volume, which became more robust when the affected animals’ data (from sheep 3 and 4) was removed from the analysis.

The stressed intravascular volume was predominantly distributed within the venous vessels with capacitance much greater than that of the left ventricle [21]. Our model involved investigation of compliance during immediate post-mortem period thus eliminating compounding influence of variable flows and active compliance normally present within multiple cardiovascular compartments of the live animal [22]. We also observed a return of ventricular fibrillation or even occasional coordinated ventricular contractions following return of exsanguinated blood back into native circulatory system. The presence of catecholamines in the returned blood was likely responsible for the return of ventricular activity. These factors may also explain the weak to moderate correlation between the mean systemic filling pressure and the left ventricular volume.

The best fit trend curves suggested a non-linear relationship between cardiovascular compliance and fluid resuscitation. The left ventricular compliance had a pronounced parabolic shape with the peak corresponding to a MSFP of 28 mmHg. The shape of the curve suggests that increase in fluid resuscitation beyond this filling pressure may result in a progressive diminutive effect on left ventricular compliance, end-diastolic left ventricular size and therefore likely progressive reduction in the Frank-Starling-regulated increase in cardiac output in a live animal. The global cardiovascular compliance appeared to also initially increase but developed the plateau trend after 30 ml/kg of fluid boluses when analysed with the entire cohort of sheep. Some decrease in global cardiovascular compliance appeared to take place following 1500 ml (30 ml/kg) of fluid boluses when sheep 3 and 4 were excluded from the analysis. Interestingly, this was the volume of fluid that raised the mean systemic filling pressure to 28 mmHg. It would be therefore appropriate to extend the volume of fluid boluses in the future study up to the volume recommended by the paediatric guidelines for management of septic shock 60 ml/kg, to determine the full effect of the extra volume on global cardiovascular compliance.

The strength of our study includes a pre-specified protocol. The study was conducted over a short inception period with high levels of data integrity. The enrolled animals were uniform in age and size. Mean systemic filling pressure was measured directly instead of by indirect estimates. Criteria were well defined and pragmatic allowing for adequate statistical power to address the primary objective that is applicable for an explorative analysis.

The limitations of this study include the small sample size and the inclusion of two sheep with protocol violations resulting in overall affected data. All sheep were receiving vasopressors, reflected by high mean values of the mean systemic filling pressure.

To our knowledge there are no previous studies investigating the relationships between mean systemic filling pressure and changes in stressed blood volume with clinically relevant intravascular fluid boluses in an ovine model. The study has translational applicability to human medicine.

Based on the pilot investigation, amendments were made to the protocol with the plan to conduct a further investigation of eight sheep. Confounding bias will be mitigated by standardising investigative techniques including modifications in CPB process to avoid future air admix and the use of crystalloid instead of the exsanguinated blood to avoid catecholamines reinfusion. The volume of crystalloid boluses will be normalized to the individual weight and administered in a total of six 10 ml/kg increments to reflect current recommendations for fluid resuscitation in adults and paediatrics. Left ventricular fibrillation and potential return to coordinated contractions will be prevented using cardioplegic solution after exsanguination. Operator-dependent errors will be mitigated by using echocardiography experts for image acquisition and analysis.

## Conclusions

It is feasible to conduct an investigation of changes in mean systemic filling pressure and cardiovascular compliance with clinically relevant intravascular fluid boluses in an ovine model.
